# An Essay on Phlegmatia Dolens, Including an Account of the Symptoms, Causes and Cure of Peritonitis Puerperalis & Conjunctiva, &c. &c.

**Published:** 1801-01

**Authors:** 


### CRITICAL RETROSPECT
OF
### MEDICAL AND PHYSICAL LITERATURE.

*An Essay on Phlegmatia Dolens, including an Account of the Symptoms, Causes and Cure of Peritonitis Puerperalis & Conjunctiva, &c. &c.* By JOHN HULL, M. D. 8vo. 370 pp. 6s. 6d. London. Bickerstaff.

WE can recommend this work as giving a very full and elaborate history and description of a disease incident to lying-in women, and commonly known by the name of *œdema lacteum*, or white leg.—The subject is treated of in the following order:

"1. The *literary history* of the disease is given, together with a *review of the different theories*, that have been formed concerning it.

"2. The *medical history* is given, and the *causes* and *cure* considered.

"3. The *analogy* of this disease with *Puerperal Fever* (Peritonitis *puerperalis* Ill. Frank.) and *Rheumatism* is pointed out.

"4. The *characters* and *synonyms* of the genus Phlegmatia with its species is presented, and the proper place assigned to it in the nosological system of the late Dr. Cullen."

In the literary history, which occupies 130 pages, the author has given copious extracts, and has frequently copied whole cases from every writer since the days of Hippocrates, who has mentioned this complaint; and we should be inclined to bestow some praise on this part of the work, as an useful epitome for those who wish to be informed of what has been written on the subject, if it was not sullied with a degree of asperity, and an unnecessary attack upon Dr. Ferriar, a gentleman of acknowleged eminence in his profession, and whose talents, as a man of letters, are of the highest order.

It is painful and vexatious to observe this contentious disposition so frequently breaking the bounds of moderation and urbanity.

"*Rest — rest, perturbed Spirit.*"

The author has illustrated the medical history of the disease by several cases, and amongst the exciting causes he enumerates, "1st. Contusions, or violent exertions of the lower portions of the abdominal and other muscles inserted into the thighs and pelvis, and of the muscles of the inferior extremities and contusions of the cellular texture connected with these muscles, during a tedious labour.—2dly. The application of cold and moisture. — 3dly. Suppression or diminution of the lochia, and of the secretion of milk. 4thly. Food taken in too large quantity and of a too stimulating quality, especially when the patient does not give suck. — 5thly. Standing or walking too much before the arteries and veins of the lower half of the body have recovered sufficiently from the effects of of the diftention which exifted during the laft months of pregnancy."—The author believes the proximate caufe "confifts in an inflammatory affection, producing fuddenly a confiderable effufion of ferum and coagulating lymph from the exhalants into the cellular membrane of the limb."

"I am induced to offer, by way of recapitulation, the following concife account of my notion of the formation, effence, &c. of Phlegmatia *dolens*: A patient, predifpofed to the difeafe, being fubjected to the action of fome, or all of the exciting caufes above-mentioned, the balance of the circulation is changed and an unufual determination of blood to the lower part of the trunk, or to one or both of the inferior extremities takes place. Hence the arteries of the part more particularly affected, are ftimulated to increafed action; inflammation is produced, and a confiderable quantity of ferum and coagulating lymph is effufed from the exhalants into the cellular membrane, which moderates the inflammation and prevents it from extending to the outer furface of the cutis. The inflammation and fwelling are fometimes more limited, but, in general, they (fooner or later in different patients) are diffufed over the whole of one limb, and not unfrequently the other lower extremity in a few days becomes affected in a fimilar way.—Whilft thefe appearances are obferved in the limb, the whole fyftem exhibits fymptoms of confiderable pyrexia; and thefe febrile fymptoms, in many inftances, precede the topical affection. After fome time, the fymptoms of pyrexia and of topical inflammation fubfiding, the general health of the patient begins to improve, and the fwelling of the limb, inftead of being tenfe, elaftic, hot, exquifitely tender and painful when touched or moved, as before, becomes of a natural heat, is ftiff and numb rather than painful or tender, yields more eafily to the finger, and retains the impreffion for a confiderable time; in fhort, the intumefcence now approaches more and more to Anafarca or Œdema, and, for the moft part, gradually difappears.—The difeafe, occafionally, has a different termination, as has already been related.

"The edematofe ftate of the limb towards the decline of the complaint may be eafily accounted for, upon the fuppofition that the power and action of the abforbents is totally unaffected; for, after the exhalants have been fo far over-ftretched as to pour out ferum and coagulating lymph, we may naturally expect, that they will be a confiderable time before they recover their tone, and be as much contracted as in their natural ftate; confequently, till that period arrive, a larger quantity of ferofity, efpecially when the patient is in an erect pofition, will be effufed from them into the cellular membrane, and accumulated in the limb, unlefs we prevent it by bandaging the limb, or increafing the action of the abforbents. From this view of the difeafe it appears, that the whole of the phenomena may be fatisfactorily accounted for independently of any primary affection of the lymphatic fyftem of the limb. Whilft, on the other hand, they cannot be explained independently on an original affection of the fanguiferous fyftem.

"In

### " SECTION THIRD.

" *Of the Cure of Phlegmatia Dolens.*

" In this complaint there are three periods, which require different modes of treatment, and which are more or lefs diftinctly marked, both with refpect to the fyftematic and topical affections, in different cafes. Hence, in delivering the method of cure, it becomes neceffary to advert to thefe three ftages.

" In the firft, which may be ftiled the *inflammatory phlogiftic*, or *fthenic period*, the affection of the fyftem for the moft part confifts in an inflammatory diathefis, or increafed tone and action of the heart and arteries, both with refpect to frequency and force; and the affection of the limb confifts in a ftill more violent action of the arteries diftributed to it, producing effufion and an extreme degree of tenfion of the fkin.

" For the removal of the fyftematic affection, the indications are,—

" 1ft. To diminifh the quantity of the circulating fluids.

" 2dly. To remove, or moderate thofe irritations, to which the body is almoft conftantly expofed.

" 3dly. To employ fedatives.

" 4thly. To employ remedies, which determine to the furface.

" The 1ft indication is fulfilled by blood-letting, purgatives and emetics.—Emetics are ufeful alfo as revulfives and by determining to the furface.

" The 2d indication is fulfilled by avoiding exercife of the mental faculties; by keeping the body quiet, and conftantly in an horizontal pofition; by ufing a low vegetable diet, and diluting acidulated liquids; by keeping the bed-chamber cool, and avoiding too great a quantity of bed-cloaths, and by keeping the bowels gently open.

" The 3d indication is fatisfied by employing remedies, which directly diminifh the action of the heart and arteries, as digitalis, acids, &c.

" The 4th indication is anfwered by employing neutral falts, antimonial preparations and ipecacuanha, either fingly, or conjointly, and ftill more completely, by combining opium with one or more of thefe remedies, and by the ufe of the warm bath.

" For moderating the violent action of the arteries of the parts affected, local blood-letting by means of leeches, and blifters or rubefacients are to be employed. For relieving the tenfion of the fkin, emollient fomentations, liniments and ointments are to be applied: punctures, or incifions, though fometimes employed with this view, are rarely very efficacious, on account of the matter coagulating very foon after its effufion.

" § 2.

" In the third, which may be named the *antiphlogiftic*, or *afthenic period*, the fyftematic affection confifts in univerfal debility, in a diminution of the tone and action of the fanguiferous fyftem, and in in an impoverifhed ftate of the blood.—The local affection appears to confift in a relaxation of and increafed effufion from the exhalants of the limb, and it is not improbable, that the action of the abforbents is fometimes diminifhed.

"For the cure of the affection of the fyftem, the indications are:—

"1ft. To increafe the general ftrength and the tone and action of the heart and arteries by bitters, tonics, ftimulants and exercife, efpecially in a carriage.

"2dly. To improve the ftate of the blood by a nutritious diet, adapted to the powers of the digeftive organs.

"For removing the topical affection, the indications are,

"1ft. To leffen the effufion of fluid from the exhalants; which may be effected partly by the remedies ufed for the cure of the fyftematic affection, and partly by the application of a tight bandage, by the cold bath, or cold water, frefh or falt, dafhed upon the limb; and by avoiding too much ftanding, or walking.

"2dly. To increafe the action of the abforbents of the limb; which is to be done by the application of heat, blifters, electricity and other ftimulants: by friction with the hand, or a flefh-brufh; by digitalis, fquill, mercury, carbonates of foda, potafh and ammoniac, &c.

"It is not fufficient, that the abforbents take up what is daily effufed by the exhalants in this ftage of the complaint; this will merely render the fwelling ftationary. It is neceffary that they abforb confiderably more, in order that the lymph and ferum, accumulated during the inflammatory ftage, may be completely removed, and the limb return to its natural fize.

"§ 3.

"In the SECOND, or *intermediate* PERIOD, the fyftematic affection is to be treated by the remedies pointed out as fulfilling the the 2d, 3d, and 4th indications in the 1ft ftage: But thefe are not to be employed fo rigoroufly.—The topical affection is to be remedied by gently ftimulating liniments, ointments, &c."

Before we proceed to the reft of the work, we take this opportunity of mentioning an application for this difeafe, which has been ufed with invariable fuccefs, for feveral years, at one of the beft regulated lying-in hofpitals in London, and where our readers will readily fuppofe, from the great number of women annually delivered there, that inftances of the *œdema lacteum*, or *phlegmatia dolens*, are not very uncommon. It is the practice at that hofpital, to apply flannel well foaked in hot vinegar to the groin of the affected limb, and to renew it frequently; and we are well affured that no other remedies beyond thofe neceffary to keep the bowels regular are ever ufed, notwithftanding the fever, pain, &c. which commonly accompany this difeafe.—The author next points out the connection and analogy fubfifting betwixt *phleg. dolens* and *peritonitis puerperalis* in their hiftories, pre-difpofing caufes, exciting and proximate caufes, and their methods of cure. Of the fymptoms of the latter difeafe he has given a good defcription, and and the following paſſage will give the author's opinion on its infectious nature, and identity with *Typhus,* neither of which he believes it is allied to.

"As far as my obſervation goes, Peritonitis *puerperalis* is not infectious. I have never ſeen a caſe, wherein I had reaſon to ſuppoſe, that the effluvia ariſing from the patient, produced puerperal fever, or typhus, or any other diſeaſe in another perſon, either directly or indirectly. The diſeaſe in queſtion frequently ariſes, where there is not the leaſt foundation for a ſuſpicion that infection has been applied. And I have, in more than one inſtance, delivered a patient in an advanced ſtage of typhus, without any ſymptoms ſupervening, that indicated an inflammatory affection of the peritonæum."

In the method of cure, the reader will perceive that no ſpecific plan is recommended, but the remedies are adapted to each particular ſpecies and habit.

"Some practitioners are, I believe, of opinion, that every caſe of a particular diſeaſe ought to be treated in an uniform manner, and hence they do not give themſelves the trouble of adapting remedies ſpecially to the different caſes that occur. Of this claſs, ſome probably may have excluſively adopted M. Doulcet's, and others Dr. Gordon's method of treating puerperal fever, according as they happened to become acquainted with one before the other, or to have an averſion to, or predilection for, the uſe of the lancet. If this be the caſe, theſe practitioners would do an eſſential ſervice to the public, by giving a full account of their ſucceſs. If this were done, I ſuſpect, that from the alleged facts it might be proved, that neither plan ought to be indiſcriminately adopted to the excluſion of the other.

"From what I have ſeen of this complaint, I am of opinion, 1ſt, that an emetic, adminiſtered early, will cure the diſeaſe in many caſes; 2dly, that general blood-letting, and briſk purging, may, in other caſes, be employed freely with ſucceſs; 3dly, that theſe two methods may, in ſome inſtances, be advantageouſly combined; 4thly, that in ſome caſes the diſeaſe may be cured without employing either general blood-letting, emetics, or briſk cathartics; 5thly, that other remedies, which have been neglected as uſeleſs and unneceſſary, or condemned as hurtful, may be joined with Doulcet's and Gordon's plans with very great advantage.

"With this impreſſion on my mind, I enter upon the conſideration of the cure of puerperal fever, although the plan I have to recommend is neither ſimple, nor uniform, nor nearly approaching to infallibility.

"The indications of cure, which ariſe in puerperal fever, reſpect either the ſyſtematic or abdominal affection. Thoſe which belong to the former head, are,

"1ſt. To moderate the action of the heart and arteries.

"2dly. To ſupport the action of the heart and arteries.

"3dly. To prevent, or correct, the putreſcency of the fluids.

"4thly. To palliate, or remove, urgent ſymptoms.

"Thoſe

"Thoſe which relate to the latter head, are,

"5thly. To moderate and ſubdue the increaſed action of the peritoneal blood-veſſels, and thereby prevent the miſchief ariſing from extenſive adheſions, from the effuſion of fluids into the abdomen, from ſuppuration and gangrene.

"6thly. To promote the abſorption of the effuſed fluids.

"7thly. To favour the diſcharge of pus when formed."

The laſt chapter treats on the character, ſynonyms, deſcription, and diagnoſis of the *genus phlegmatia* and its ſpecies, and is a ſpecimen of the noſological abilities of the author.

